# Supplementary material for: Perceptions of Quality of Interprofessional Collaboration, Staff Well-Being and Nonbeneficial Treatment: A Comparison between Nurses and Physicians in Intensive and Palliative Care
Source: Healthcare (Basel). 2024 Mar 7;12(6):602. doi: 10.3390/healthcare12060602 (PMC10969805; doi:10.3390/healthcare12060602)
Supplement: Supplementary file 1 [file healthcare-12-00602-s001.zip › healthcare-2866221-supplementary.pdf]

Online Supplement: Perceptions of quality of interprofessional collaboration, staff well-being and nonbeneficial treatment: A comparison between nurses and physicians in intensive and palliative care

Supplemental Figures

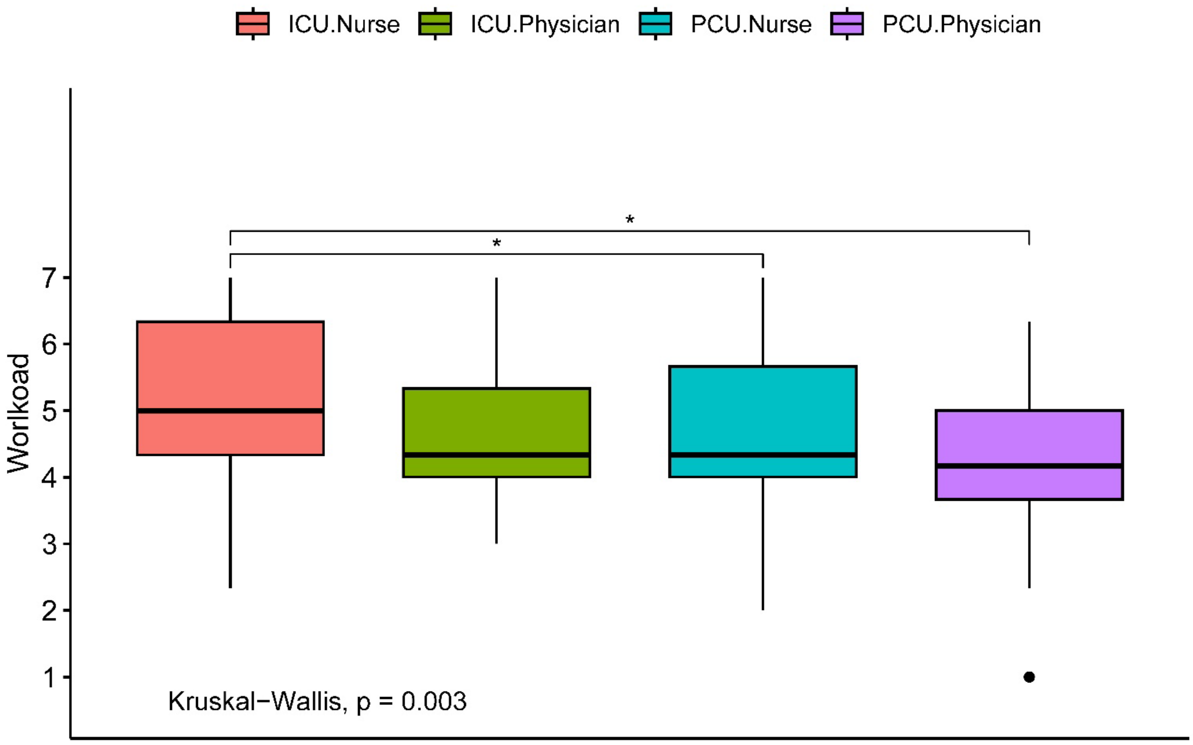

Figure S1. Comparison of perceived workload between intensive care and palliative care nurses and physicians. Tests of differences between groups were performed using the Kruskal-Wallis test. Comparisons between individual groups were performed using Wilcoxon rank sum tests adjusted for multiple comparisons by the Holm method (significance level: **** $p \leq 0.0001$, *** $p \leq 0.001$, ** $p \leq 0.01$, * $p \leq 0.05$, no parenthesis: not significant).

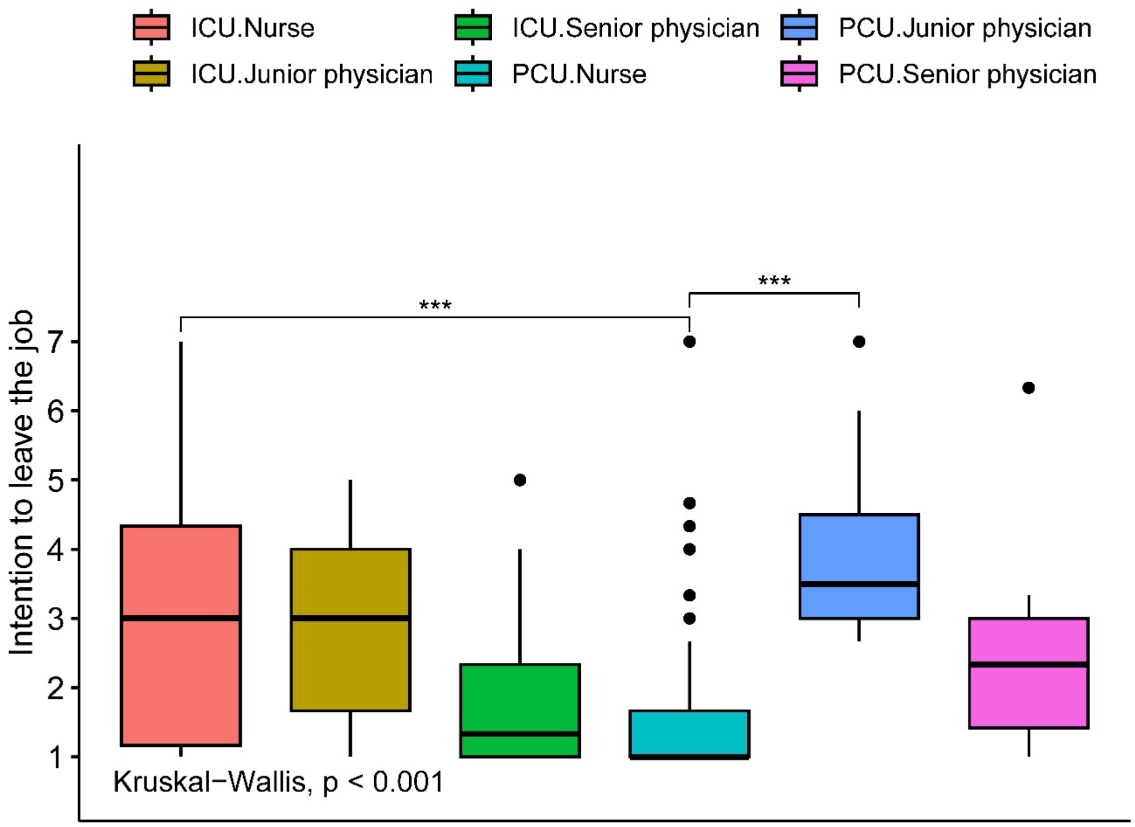

Figure S2. Comparison of intention to leave the job between intensive care and palliative care nurses, junior, and senior physicians. Tests of differences between groups were performed using the Kruskal-Wallis test. Comparisons between individual groups were performed using Wilcoxon rank sum tests adjusted for multiple comparisons by the Holm method (significance level: **** $p \leq 0.0001$, *** $p \leq 0.001$, ** $p \leq 0.01$, * $p \leq 0.05$, no parenthesis: not significant).

Supplemental Tables

Table S1. Items of the questionnaire

	Intensive care, *n* = 72	Palliative care, *n* = 72	P value	Cronbach's alpha intensive care	Cronbach's alpha palliative care
**Workload** (1:totally disagree – 7:totally agree)				0.82	0.74
I have to work very hard.	6 [4, 6]	5 [4, 6]	0.075		
I am asked to do an excessive amount of work.	5 [4, 6]	4 [3.75, 5]	0.007		
I don't have enough time to get my work done.	5 [4, 6]	4 [4, 5]	0.025		
**Overall quality of collaboration** (1:very low – 5:very high)				0.81	0.73
Describe the quality of collaboration and communication you have experienced with…Nurses	4 [3, 4]	4 [4, 5]	≤0.001		
…Head Nurse	3 [2, 4]	5 [4, 5]	≤0.001		
…Residents	4 [3, 4]	4 [3, 4]	0.135		
…Attendings	4 [3, 4]	4 [3, 5]	0.027		
…Consultant Physicians	3 [2, 4]	3 [2.75, 4]	0.833		
…Occupational/Physio-therapists	4 [3, 4]	4 [4, 5]	0.002		

…Psychologists/Social Workers	3.5 [3, 4]	4 [4, 5]	≤0.001		
**Collaboration about Care Decisions** (1:totally disagree – 7:totally agree)				0.91	0.88
Nurses and physicians plan together to make decisions about care for patients.	4 [3, 5]	6 [4, 6]	≤0.001		
Open communication between physicians and nurses takes place as decisions are made for patients.	4 [3, 5]	6 [4.75, 6]	≤0.001		
Physicians and nurses cooperate in making decisions regarding patient care.	4 [3, 5]	6 [5, 6]	≤0.001		
In making decisions about patient care, both nursing and medical concerns are considered.	4 [3.75, 5]	6 [5, 6]	≤0.001		
**Inclusive leadership by attendings** (1:totally disagree – 7:totally agree)				0.92	0.94
Attending physicians of this unit encourage residents to take initiative in patient care.	5 [4, 6]	6 [4, 7]	0.002		
Attending physicians of this unit ask residents for input.	4 [4, 6]	6 [4, 6]	0.004		
Attending physicians of this unit do not value opinions of residents equally.	4 [4, 6]	6 [4, 6]	0.003		
Attending physicians of this unit are open to discuss differing opinions with residents.	4 [3, 6]	6 [4, 6]	0.007		
**Inclusive leadership by head nurse** (1:totally disagree – 7:totally agree)					

The head nurse of this unit encourages nurses to take initiative in patient care.	4.5 [3, 6]	6 [6, 7]	≤.001	0.91	0.9
The head nurse of this unit asks nurses for input.	4 [3, 6]	6 [6, 7]	≤0.001		
The head nurse of this unit does not value opinions of nurses equally.	4 [2, 6]	7 [6, 7]	≤0.001		
The head nurse of this unit is open to discuss differing opinions with nurses.	4 [3, 6]	7 [6, 7]	≤0.001		
**Nonbeneficial treatment** (1:never – 6:very often)				0.93	0.92
For the patients you treat on your unit how often do you perceive that…a fatally ill patient receives unnecessary diagnostic tests.	4 [4, 5]	4 [2, 4.5]	≤0.001		
…a patient receives too much life-sustaining treatment given his prognosis.	5 [4, 5]	3.5 [2, 4]	≤0.001		
…continued life-sustaining treatment unnecessarily prolongs the suffering of a patient.	5 [4, 5]	3 [2, 4]	≤0.001		
…intensive resuscitation is done on a patient that only delays that patients' near death.	4 [3, 5]	1 [1, 2]	≤0.001		
…decisions for limiting life-sustaining treatments are unnecessarily delayed.	4 [4, 5]	3 [2, 4]	≤0.001		
**Emotional exhaustion** (1:never – 6:very often)				0.9	0.88
I feel burned out from my work.	4 [3, 4]	4 [3, 4]	0.433		

I feel emotionally drained from my work.	3.5 [3, 4]	4 [2.5, 4]	0.493		
I feel fatigued when I get up in the morning and have to face another day on the job.	3 [2, 4]	4 [3, 4]	0.933		
Working a whole shift is really a strain for me	3 [2, 4]	3 [2, 4]	0.277		
I feel used up at the end of the workday.	4 [3, 5]	4 [3, 4]	0.09		
**Intention to leave the job** (1:totally disagree – 7:totally agree)				0.79	0.81
I plan on leaving my job within the next year.	1 [1, 4]	1 [1, 2]	0.117		
I have been actively looking for other jobs.	1 [1, 4]	1 [1, 2]	0.036		
I want to remain in my job.	5.5 [4, 7]	7 [5, 7]	0.009		

Descriptive statistics given as *median* [*1st quartile*, *3rd quartile*]. Significance testing by Wilcoxon rank sum test.

Table S2. Characteristics of hospitals and units

	Hospital A	Hospital B	Hospital C	Hospital D	Hospital E	Hospital F
**Hospital**						
Level of care	Primary care	University hospital	University hospital	Primary care	University hospital	Primary care
Hospital beds (*n*)	429	800	1324	330	1350	186
**Intensive care unit**						
Type of unit	Mixed	Surgical	Surgical	Mixed	Surgical	Mixed
Medical leadership	Anesthesiology	Anesthesiology	Anesthesiology	Anesthesiology & Internal medicine (cardiology)	Anesthesiology	Anesthesiology
Beds (*n*)	10	25	50	10	58	7
Physicians (*n*)	12	52	55	13	44	5
Nurses (*n*)	31	93	178	49	165	33
Occupational/physiotherapists integrated in the team	Yes	Yes	No	Yes	No	No
Psychologist integrated in the team	No	No	Yes	No	Yes	No

Social worker integrated in the team	No	No	No	No	No	No
**Palliative care unit**						
Medical leadership	Internal medicine (oncology)	Internal medicine (oncology)	Anesthesiology & Internal medicine (oncology)	Internal medicine (oncology)	Anesthesiology	Anesthesiology
Beds (n)	10	10	10	12	12	7
Physicians (*n*)	2	4	13	2	4	4
Nurses (*n*)	10	10	18	22	15	9
Occupational/physiotherapist integrated in the team	Yes	Yes	Yes	Yes	Yes	Yes
Psychologist integrated in the team	Yes	Yes	Yes	No	Yes	No
Social worker integrated in the team	Yes	Yes	Yes	Yes	Yes	Yes

Table S3. Balance measured by standardized differences before and after matching.

	ITS, N = 313	Palli, N = 79	Standardized difference	ITS, N = 72	Palli, N = 72	Standardized difference
Hospital A	29 (9.3)	9 (11.4)	-0.07	9 (12.5)	9 (12.5)	0
Hospital B	21 (6.7)	9 (11.4)	-0.16	9 (12.5)	9 (12.5)	0
Hospital C	117 (37.4)	23 (29.1)	0.18	23 (31.9)	23 (31.9)	0
Hospital D	36 (11.5)	19 (24.1)	-0.33	13 (18.1)	13 (18.1)	0
Hospital E	96 (30.7)	9 (11.4)	0.49	9 (12.5)	9 (12.5)	0
Hospital F	14 (4.5)	10 (12.7)	-0.3	9 (12.5)	9 (12.5)	0
Job role						
Nurse	232 (74.1)	53 (67.1)	0.15	50 (69.4)	49 (68.1)	0.03
Head nurse	6 (1.9)	6 (7.6)	-0.27	3 (4.2)	3 (4.2)	0
Resident	52 (16.6)	9 (11.4)	0.15	8 (11.1)	9 (12.5)	-0.04
Attending	23 (7.3)	11 (13.9)	-0.21	11 (15.3)	11 (15.3)	0
Sex: Female	209 (69.9)	55 (74.3)	-0.1	49 (71)	48 (71.6)	-0.01
Age ≥ 40 years	77 (25.9)	53 (73.6)	1.08	45 (65.2)	46 (70.8)	0.12
Medical experience ≥ 5 years	189 (66.8)	65 (91.5)	0.64	58 (90.6)	58 (90.6)	0

Descriptive statistics given as *n* (*%*)
